# Abstract of the Proceedings of the Buffalo Medical Association

**Published:** 1862-05

**Authors:** Julius F. Miner

**Affiliations:** Secretary


ART. II.—*Abstract of the Proceedings of the Buffalo Medical Association.—Retiring President's Address.*

ANNUAL MEETING, April 1, 1862.

The minutes of the last meeting were read, and after being corrected, to say, that Dr. Gay, and not Dr. White, suggested the use of solution of per sulphate of iron in menorrhagia, giving 12 drops 3 times daily, and not every 4 hours, as reported, were adopted as published.

The reports of Secretary, Treasurer and Librarian were read, accepted and voted to be placed on file.

ELECTION OF OFFICERS.

| | |
|---|---|
| JAMES P. WHITE, M. D., | President. |
| HORACE M. CONGAR, M. D., | Vice President. |
| JULIUS F. MINER, M. D., | Secretary. |
| J. B. SAMO, M D., | Treasurer. |
| WILLIAM GOULD, M. D, | Librarian. |

The retiring President, Dr. Gay, then read the following address:

MR. VICE PRESIDENT AND GENTLEMEN OF THE BUFFALO MEDICAL ASSOCIATION:—

You have now entered upon the eighteenth year since the organization of the Buffalo Medical Association, and the seventh year since its incorporation. The meetings have been regularly held on the first Tuesday evening of each month with an average numerical attendance encouraging, for the most part comprised of practitioners in good and regular standing, of the city, while occasionally, physicians from abroad have been invited to seats, have participated in its proceedings and contributed to its interests. The Association has been a fruitful source of improvement to all who have availed themselves of its advantages, the proceedings have been faithfully reported and published in the *Buffalo Medical Journal*, thus enlarging the sphere of the Society's usefulness, and many of its most able papers have from time to time been quoted by other medical periodicals at home and abroad, and referred to with much consideration in the transactions of the State Medical Society.

My immediate predecessor, on retiring from the chair, presented to the Association a synopsis of the proceedings, extending over a period of sixteen years, and indeed embracing the proceedings of former organizations from the year 1831 to the year 1861 inclusive. You need not be told that this duty was performed with much ability and fidelity. We thus have the proceedings embodied in permanent form—published in the *Journal*, which, while they will remain a most valuable record of the names and practice of the earlier and later physicians—will stand as a monument to the patient labor of the compiler.

It is, gentlemen, just cause for congratulation that the Association has been attended with much usefulness and success. It is good for physicians to meet together, to confer, one with another, to interchange views and opinions and to foster friendly and fraternal relations. It is another cause of congratulation, that the profession of the city, which was formerly divided, either for real or imaginary causes, is now united and harmonious. The voice of detraction is silent and unity of purpose with common ambitions and aspirations prevail. Thus united and strong we may the more efficiently combat the enemy to which all flesh is heir and successfully fulfill our sacred mission.

The war in which we are engaged, caused by the wicked and unwarrantable rebellion of one section of our common country against the other section, and the attempt of the government to maintain itself intact, to preserve the precious boon transmitted to us by the fathers of the great Republic, by putting down and subduing the insurgents, has called into the tented field many members of our profession. I am happy to assure you that Buffalo has furnished her quota, both for the army and navy of the regular and volunteer service. Patriotic and in many instance self-abnegating, these, our brothers, have gone with alacrity in response to their country's call, to fields of honor and duty. Let us cherish the hope that, their lives may be preserved to return from the "sacred soil," again to be with us, to enliven and enrich our proceedings with the detail of personal experiences of camp life and military surgery.

Physicians, as a class can fully appreciate, I think, the import of the term rebellion. We have had always to guard against and combat it. Science is the firm basis on which medicine as well as government rests. She is the corner stone of the edifice, but parties have arisen up at various times and places during the whole history of medicine with their innovations and inventions, assaying to overthrow and subvert medicine, to revolutionize it and to substitute a system of their own.

These medical rebels—assuming different disguises—have with much apparent ability and great subtlety, practiced upon the credulity of the people and summoned around them many supporters and advocates.—But thus far these medical seceders have utterly and ignominiously failed, their new principles and theories proving ephemeral and their efforts abortive. We have now the cheering hope that these political seceders and innovators will soon meet with a like inglorious end.

It has been said, that "medicine is not the offspring of the human intellect, she is the daughter of time, necessity brought her to light, experience perfected her." The object of medical science is to prevent and cure disease and to prolong life. The science and the art have been diligently studied and earnestly practiced from the earliest ages to the present time. Its propriety and necessity are apparent. You cannot look out upon the world without being arrested at every view with the lamentable fact, that disease benign or malignant, disease—either acquired or transmitted—disease all-pervading—is the common heritage of the race. The relative proportion of disease and health, I suppose, has never been ascertained. But so much the physician knows—that when he sees an individual in the full enjoyment of a perfect and harmonious performance of all the functions of the economy, constituting that physiological condition denominated health—he sees one who is an exception to that opposite condition which constitutes the rule.—I embrace in my definition of disease not only those who are fit subjects for medical treatment, but those also who manifest but the slightest departure from that normal condition which we call health, and who are not fit subjects for medical treatment. When there is superadded to the diseases that are acquired, those that are constitutional—the latter being transmissible from generation to generation—we obtain a clearer comprehension of the amount of disease in the world. If disease existing in one generation is permitted to propagate and perpetuate itself in the absence of appropriate medical appliances, we are to presume that the succeeding generation will suffer in consequence of such neglect. In other words, a generation of invalids, like one of inebriates, begets its like in the next.

This pathological condition of our race is more apparent to the physician than to others. Who but the physician can affirm as the knowledge is revealed to him by the stethescope, that disease is making its insidious approach or is so far advanced that the process of undermining a strong constitution, has already begun. The victim himself has not the knowledge, for he has yet experienced none of the pains and inconveniences incident to its advance. Tell me, how many in the active business of life are able to know that structural changes are going on within them, converting healthy lung tissue into tubercle, or an artery into an aneurism or the valves of the heart into bone,

> "Whose hearts, like muffled drums,
> Are beating funeral marches to the grave"

Medical science, alone, is able to decide and say when disease is structural or only functional and this knowledge often becomes a source of grief to the physician who knows, what his friend is not presumed to know, until he is informed, that he possesses a malady which must inevitably terminate and perhaps suddenly, that friendship, by terminating the life of the friend.

There is indeed more disease and less health in the world, than is usually dreamed of in the philosophy of the casual observer.

Upon such data we may legitimately conclude that disease is rife in the land, everywhere present and no where absent, and like original sin itself, is the *normal,* and health the *abnormal* condition of man. Upon this was founded the idea that, the race was in process of extermination and was destined at some remote period, actually to disappear from the face of the earth, giving place to a better and superior race. But there is a class of pseudo-philosophers who look upon our profession with great disfavor. By them, it is regarded as a useless excrescence upon the body-politic, destined in time to slough off. Were it needful to say more to persuade such, that the profession is at least a necessary evil, I might quote from the sacred volume, but to them such testimony would be valueless.

Solomon, the wise man, is reported to have said : "Honor the physician with the honor due unto him for the Lord hath created him, from the Most High cometh healing and he shall receive honor of the King." It is also written : "Give place to the physician for the Lord hath created him." Again : "The Lord hath created medicines out of the earth and he that is wise will not abhor them.

I might also cite the opinions of the great and good of the earth who have personal reasons for testifying in its behalf, indeed we are surrounded by a cloud of witnessess all concurring in opinion, to the propriety and necessity of the healing art. The original sin which entered and poisoned the world, the cause of all our woes, seeming to grow with our growth and strengthen with our strength, the constant and repeated infraction of the laws of our being always followed by the penalty, loudly proclaim its necessity.

Why Adam could not have been content and happy in the beautiful garden of Eden, surrounded by all that was lovely in nature, is a mystery.—Had he foreseen an infinitesimal amount of the misery and woe his act was to entail upon his posterity, I am sure he would have regarded and obeyed the Divine injunction. Such is our fallen estate. With the Providence which has so ordained our physical condition we are wise to have no controversy, afflictive as well as merciful providences are but means to the Divine end. The same All-wise and Beneficient Creator who gave us being has placed at our disposal the means, by the judicious use of which, we may go forth armed for the contest. The field of duty is an uninviting one, the pathway is not strewn with fragrant flowers, but human woe and misery meet us at every step.

The grand army of physicians must go forth not like the armies in battle to be men killers, but curers, and amid malaria and contagion, armed with henbane, pœnia, the cinchonia and night shade, give battle and attempt to achieve honor and victory

To mitigate human pain and woe and to assuage the physical ills incident to human life, is the province of the physician and this is no mean avocation, but he who enters its ranks must leave all hopes of personal ease, emolument and renown behind. Success will not depend upon the number of patients nor amount of money treasured, for if these were the true criteria of success, the legitimate practitioner could never hope to approximate so nearly the standard as the merest pretender. But to the faithful and laborious service rendered the profession—the promotion of its progress, his guardianship of its integrity and to the able ministrations of its kindly offices, is he to look for success. It is conceded by all that a greater advance has been made in the science of medicine during the present century than at any other former period of its history. This is owing to a tripple cause. We possess a greater abundance of knowledge, have acquired superior modes of investigating disease and have an improved materia medica.

First. A greater abundance of knowledge has been attained from a record of the labors of the past, appropriating the observations of value on the one hand, of those who have lived and labored in the past and avoiding the into errors, on the other.

As an instance in point of the errors of the past and their causes, I may remark that, physicians in the middle ages were invariably priests, whom a canon of the church forbid to shed blood, therefore surgical operations fell into the hands of an inferior class of barber surgeons.

Ambrose Paré who commenced his carreer as a barber surgeon, and who afterwards gave great impulse to surgery, was accustomed considering wounds received from fire-arms poisonous, to cauterize their track with boiling oil.

On one occasion while serving as surgeon in the French Army, Pare's supply of oil failed him, He could not sleep for anxiety, but when morning came he found that those who had not been cauterized were better than those who had been and the observation led to a revolution in practice.

A greater abundance of knowledge results from the greater perfection and certainty of the sciences immediately pertaining to medicine and from the late developement of the sciences collateral to medicine. Experimental physiology which is now prosecuted with an energy truly surprising, has contributed greatly to the advance of medicine.

Histology brings her offerings and lays them on the altar, fit token of her devotion to medicine; the microscope has brought to light a new world and is unfolding the mysteries of our being. Chemistry has been made almost a new science by her brilliant discoveries, while it unfolds the intimate structures of the solids and fluids of the body, shedding light concerning the functions in health and disease, it analyses the drugs employed in the treatment of morbid conditions and detects the minutest atom of any irritant poison taken for criminal purposes.

Botany also has been pressed into service. No physician in our time feels that his medical education is complete without a knowledge of at least the plants indigenous to his country. Not one of the collateral sciences is so well adapted to impart strength to the faculty of discrimination or discipline to the mind as botany.

To the other qualifications of a physician, add that of a good botanist and you can be no longer in doubt as to his being a good physician. I may add that during the process of analyzation we become first acquainted with the physical properties of the plant and at once pass imperceptibly within the domain of medicine and begin our inquiries in relation to its medical properties, thus showing how intimately interwoven are the sciences and how by a process of imbrication one science laps upon another.

Second. We possess superior modes of investigating disease. It is no less painful to the physician to be in doubt concerning the nature of the case he is called upon to treat, than it is unfortunate to his patient. To arrive early in the first stage of disease to a correct diagnosis is all important to a rational mode of treatment. It was reserved for Laennec, by his great dicsovery of ausculation and percussion to superadd to the rational, the physical signs of disease thus changing the whole face of medicine and giving it a degree of certainty which before seemed hopeless. By the aid of this discovery, improved and brought more nearly to perfection by much labor and study, Dr. Austin Flint, an early member of this Association, has been able to give to the world a work on the diseases of the heart which stands without a rival in any language.

Third. We have an improved materia medica. The discovery of the active principles of the various drugs, the use of which is more certain and administration, less offensive, is an essential manifestation of advancement. The alkaloids, morphia in opium, quinia in peruvian bark, strychnea in nux vomica and the addition of numberless other ingredients, have enabled us to prescribe in a more elegant and potent form different remedial agents. In addition to the superiority of the different preparations now in use, many new and valuable ones have been discovered.

The veratrum veride, which is found to reduce the volume and frequency of the pulse, is now used in place of the lancet in many instances, the perchlorate of iron as a styptic, the persulphate of iron both as a styptic and for internal administration in anemia, menorrhagia, leucorrhoea &c., and many other remedies which time will not permit me to speak, are some of the discoveries which give indication of the advance in this department of medicine. To the causes of advance already enumerated we might add without limit. The improvement in the various branches of medical science cannot but be attended with a corresponding benefit to the sick and to society at large. In proof of which let a few facts be submitted: Victories of medical science over disease and death may be found recorded upon almost every page of the history of medicine, and the trophies of victory and success constitute our pride and boast. They carry within them to the minds of the most incredulous, unless so blind as they that would not see the conviction, that medicine is not all in vain and that its advance is in direct ratio to the advance of education and civilization. You will permit me therefore to make a rapid survey of the domain of medicine and to point out, in brief, some of the more important results consequent upon the combined labors of medical men.

It was a bold step of Mondini, about the year 1300, to dissect the bodies of two females, which he did with the exception of the head, which he dared not open for fear of committing a mortal sin. He afterwards published an anatomical description of the body, illustrated with wood cuts, which answered the purpose of physicians for nearly two hundred years. Before the close of the fifteenth century human dissections were prosecuted at Bologna, and in the year 1543 Versaleus published his great work on Anatomy. The prejudices against human dissections soon thereafter were mitigated, and the invention of the art of printing and engraving served to spread abroad and perpetuate the discoveries made. Early superstitions and prejudices have now disappeared and dissections are legalized.

Toward the end of the fifteenth century syphilis made its appearance and was more virulent and fatal than any other disease. Fifty thousand are said to have died of it in one year in Naples. An antidote was found in mercury and places it entirely within our control. The scurvy which numbered its victims by the thousands, at once the terror and foe to the marines, at length found its appropriate remedy.

Harvey in the year 1616 discovered the circulation of the blood, and in 1661 Malpighi by the aid of the microscope showed the course of the globules of the blood in the smaller vessels. The true theory of respiration soon followed the discovery of the circulation. In 1639 Peruvian bark is said to have been introduced into Spain by the Countess of Conchon, and its use therefrom became common throughout Europe. Before its discovery the ancients with whom malarious diseases were common, had no specific means of arresting their attacks; even mild cases of intermittents often continued for an indefinite time, and finally induced organic changes.

If anything was wanting to show the immense importance and possible influence over the civilized world of a cultivation of the science of medicine this would do it; that had the Cinchona bark been in use a few years earlier it would in all human probability have saved the life of Cromwell, and caused the history of England to have been re-written.

Until the end of the last century small pox committed the most fearful ravages, the deaths from it alone in Europe were estimated to amount to 400,000 annually, while it left many others blind or disfigured. By the discovery of Jenner of vaccination the disease is placed completely under our control, and if it still commits occasional ravages it is owing to the carelessness of individuals and the laxity of the laws.

Cholera, which at first made such fearful inroads, decimating whole districts at each visitation, has now become to a great extent manageable. The discovery and use of anæsthetics dating from 1847, is one of the greatest boons medicine has ever conferred upon suffering humanity. It enables the accoucheur to perform his sacred duties while his patient is wholly insensible to the pains of labor, and the surgeon to ply his art while his patient is lying down to pleasant dreams. More comprehensive and enlightened views of the pathology of some of the more intractable affections have led the way to reform in treatment. Puerperal fever, peritonitis and puerperal convulsions have yielded much of their terror to the more potent agency of opium. The treatment of pneumonia and phthisis is essentially modified and improved. Other forms of disease, hitherto deemed quite intractable, are now so managed and controlled as to rescue their victims from the jaws of the grim messenger.

Reliable statistics show that medical science has diminished the death rate and added from five to seven years to the average length of life. The labor in the aggregate saved through the instrumentality of the profession in saving and prolonging life, would add to the material wealth of the world an amount beyond computation. These are truly trophies of success. Such is medicine, raised to the dignity of a science by the labors and observations of the "Father of Medicine," 460 years B. C., cultivated by the bright and shining intellects of the past centuries, and transmitted to us revised and improved. Shall we be its conservators? Ours is the task to labor to perfect it—to add to and promote its usefulness, and to transmit it unimpaired, if not improved, to the generations that are to succeed us. This, I recognize as the mission of this Association. Individual effort can, if wisely directed, do much good, but combined effort can accomplish more. No immediate results may be perceptible from your associated labors, but the remote results will be certain and inevitable. Learn then to labor and to wait, for certain it is, that he who is content to wait and not to labor, will find at length that he has become what a distinguished professor very properly styles a "monumental physician" Doctors of regular medicine may, like plants, be divided into two classes, viz: phenogamous and cryptogamous doctors—the one class unfolding and exhibiting their corolla; the other refusing, or having none to unfold; the one beautifying and adorning, the other covering up and concealing; the one letting their light so shine that their works may be seen of men to be good or evil, the other putting their lights under a bushel.

But the true reason, doubtless, why each in turn does not deposit in the temple, votive tablets upon which are inscribed some account of their observations and study, is, that native modesty which underestimates the value of their contributions. The *honor* and *integrity* of the profession demand our first and foremost attention. Among so large a number it were to be expected that there would occasionally be one or more who sought personal aggrandizement at the expense and even to the detriment of his calling; should any there be, but little harm can accrue, provided they serve not as stumbling blocks to others. It is in the ranks of empiricism alone that we are to look for pretenders who grow arrogant while they revel upon the credulity of the people. Imposters will exist so long as there are people who love to be imposed upon.

Mankind, according to education, habits or peculiar mental conformation, may be divided into two classes; one class characterized by belief and stability, the other by unbelief and instability; one class governed by fixed and immutable laws, the other, having no governing principle, are tossed about by every wind of false doctrine, and hence in medical parlance are denominated quacks.

Now let me remind you that empiricism is not peculiar to medicine; it is peculiar to a *class*. In our own country there is such a strong infusion of the "Young America" element which recognizes children, men of a smaller growth, and has little or no respect for age, or regard for superiors or authority, that science is obscured and real worth unappreciated. As a logical sequence we have quacks in politics, war and trade, spiritual and legal quacks and quacks medical.

In *politics* there are on the one hand those who believe in the Divine origin of good government; are loyal and obedient citizens, and regard their constitutional obligations. On the other hand there are those who, becoming followers of false guides, rebel against the government and secede from it, and who, on the ruins of a government which has furnished them protection and numberless blessings, would rear another of their own choice.

In *war* these charlatans belong to the "Onward to Richmond" party, who, wise in their own conceit, regard science too slow, unless she perform daily some brilliant feat, would wrest, from the hands of those educated in the science of war, and who have hitherto led on our armies to victory and glory, the management of the war, and conduct it upon the basis of their superior insight, or upon what may be termed the law of empericism.

In *trade* how few are governed by the legitimate rules of trade and follow persistently and successfully their business to the end. The majority depend upon the changing wind, in all their pursuits through life.

After some considerable inquiry and observation I am enabled to affirm that quackery in business was the chief element in producing the recent financial revulsion, and that a majority of the large failures were of this class, while those who conducted their business upon a legitimate basis were enabled to stem the current and outride the storm

In *theology* one class accept the principles of the Gospel as the "rule of their faith and practice;" the other choosing rather to reject those precepts which would serve as a "lamp unto their feet and a light unto their path," become advocates of spiritualism with all its mutterings and rappings.

In *law* we have pettifoggers as well as the legally qualified practitioners. But in *medicine* the hydra-headed monster mostly thrives; cut off one head and other appears. The greater the absurdity and the more miraculous the empirical system the more converts to the system. The love of the miraculous now as in the time of the dark ages, has its hold firmly upon the people, and many are ready to believe that one person can diagnose disease as accurately by a lock of the hair as another can by his science.

The different empirical systems in vogue, if they are of no other value, become valuable in the sense that a counterfeit bank bill is valuable by proving that there is somewhere a genuine one. Medical innovations and crude schemes of medicine will doubtless in time to come, as in time past, or at least until a higher standard of education shall correct the errors of the past—have their rise, progress and decline during the brief life-time of their inventors and authors, one theory and system giving place to another theory and system, and so on to the end. But not until the granite rocks shall have moldered and crumbled into dust will *true* medicine decline.

The authors of the different empirical systems assuming to leap, like the goddess Minerva, full-armed from the head of Jove, erect their own perishable monuments of *brass*. Not so the man of science, toilsome industry builds more useful if not so splendid monuments to its patient labor.

Do you ask how best we may combat empiricism? I reply, let it severely alone, or if an attack must needs be made, avoid all pretext for a charge of persecution by making that attack by a flank movement. Go to work and promote and diffuse the sciences among the people. Where the sciences are cultivated, empiricism, like the weeds of a well cultivated garden, wlll go to decay. Science and empiricism are incompatable. They can not exist together. Who ever knew a scientific man to be a quack, or a quack a scientific man? Science then is the antidote, and we have deep interest in its general diffusion among the people. But science may be objectionable to some because she is modest. She will not permit her votaries to succeed—in the common acceptation of the term. It is, I regret to acknowledge, too often the case that the medical man of science and modesty must give way to the man of less science and less modesty.

In this connection we have reason to felicitate ourselves upon the fact of the formation in this City of a Society for the promotion of the natural sciences. The energy already manifested by its founders to place this Society upon a firm basis, and the wisdom evinced in the choice of its first presiding officer, gives promise of success. Let us extend to this new organization the right hand of fellowship.

From the foregoing we learn that medicine is a science, embracing within its folds many sciences *de facto*, in the welfare of which the people at large have much greater interest than is duly appreciated by them. For *it* and for *ourselves* we ask for no legislative ægis, but did the business world evince one-half the concern for protection against medical counterfeits that it evinces for legislative protection against counterfeit bank notes, it would demonstrate its greater love of life than of money.

In conclusion, gentlemen, let the object of your united labors be directed first and foremost to the maintenance of the honor and integrity of the profession of your choice. Let no "pent up Utica" contract your powers; cultivate medicine in all its departments. And may the tree planted by the fathers of medicine, watered and nurtured by their care, suffer no neglect at your hands. May it grow in heighth and breadth and strength until the scientific branches thereof, collectively, shall afford protection to the infirm, a refuge to the afflicted, and the fruit thereof healing to the nations.

*Voted,* That the thanks of the Society be presented Dr. Gay for his interesting Address, and a copy requested for publication in the *Buffalo Medical Journal.*

*Voted,* That Dr. Elliot of Fort Erie, C. W., be invited to participate in the proceedings of the Association.

*Voted,* That Dr. John McKinnon of Buffalo, be invited to participate in the proceedings until he can complete his membership in the Erie County Medical Society.

*Voted* to adjourn to Tuesday evening, May 6th.

JULIUS F. MINER, Secretary.

